# Pediatric clinical nurses’ role conflict and adaptation status: time for improvement

**DOI:** 10.3389/fpsyt.2026.1754467

**Published:** 2026-05-12

**Authors:** Daolin Ye, Shangju Chen, Mengya Li, Ying Yang, Ping Wu

**Affiliations:** 1 Department of Burn and Plastic Surgery, Children’s Hospital of Nanjing Medical University, Nanjing, Jiangsu, China; 2 Department of Orthopedic, Children’s Hospital of Nanjing Medical University, Nanjing, Jiangsu, China

**Keywords:** adaptation, care, clinical, conflict, management, nurses, pediatric, role

## Abstract

**Background:**

Pediatric nurses face unique clinical challenges that increase their risk of role conflict, which may adversely affect role adaptation and care quality. However, the mechanisms underlying the relationship between role conflict and adaptation, as well as targeted interventions, remain understudied.

**Objective:**

This study aimed to investigate the current status of role conflict and adaptation among pediatric clinical nurses, identify influencing factors, and explore mediating and moderating mechanisms to inform clinical practice.

**Methods:**

A cross-sectional survey was conducted at a tertiary grade A specialized children’s hospital in China. Data were collected using the Nurse Role Conflict Scale (NRCS), Nurse Role Adaptation Scale (NRAS). Statistical analyses included descriptive statistics, Pearson correlation analysis, multiple linear regression, and mediation/moderation tests using the Hayes PROCESS macro with 5000 Bootstrap samples.

**Results:**

A total of 426 pediatric clinical nurses were included. The mean scores of role conflict and role adaptation among pediatric nurses were 52.46 ± 7.55 and 85.20 ± 10.48, respectively. Role conflict was significantly negatively correlated with role adaptation (r=-0.728, P<0.001). Multiple regression identified gender, age, department, work experience, professional title, number of children, and psychological support training as independent predictors of role conflict, while gender, age, department, marital status, and psychological support training predicted role adaptation. Role cognition mediated the relationship between role conflict and adaptation (indirect effect=-0.407, 95%CI: -0.503 to -0.312), accounting for 55.91% of the total effect. Psychological support training moderated this relationship (β=0.153, P<0.001), buffering the negative impact of role conflict on adaptation. Significant interdepartmental differences were observed, with pediatric ICU and emergency nurses reporting the highest role conflict and lowest adaptation.

**Conclusion:**

Pediatric nurses experience moderate role conflict and relatively good adaptation, with multiple demographic and occupational factors influencing these constructs. The mediating role of role cognition and moderating role of psychological support training highlight the need for targeted interventions.

## Introduction

As a highly specialized field in the medical service system, pediatrics is characterized by rapid changes in children’s conditions, limited expressive abilities, and poor treatment compliance, which place higher requirements on the professional competence, communication skills, and psychological resilience of clinical nurses (1). In clinical practice, pediatric nurses are required to assume multiple roles simultaneously, such as “treatment implementer”, “health educator”, and “family supporter”: they must not only accurately perform diagnostic and therapeutic operations and closely monitor changes in patients’ conditions but also take into account the psychological comfort of children and health guidance for their families, as well as respond to the emergency handling of sudden medical events (2). This combination of multiple roles and the high-intensity, high-pressure nature of the medical environment makes pediatric nurses a high-risk group for role conflict, mainly manifested in the contradiction between professional ideals and clinical reality, the imbalance between workload and energy allocation, and the gap between families’ high expectations and limited nursing resources (3). Long-term exposure to role conflict can not only lead to job burnout and emotional exhaustion among nurses but also affect the quality of nursing services, increase the risk of medical errors, and thereby exert adverse impacts on children’s prognosis and doctor-patient relationships (4).

With the transformation of the pediatric medical model towards the “bio-psycho-social” medical model, the connotation of nurses’ roles has been continuously expanded, and their role adaptation ability has become a key factor affecting the continuity and safety of pediatric medical services (5). Currently, the pediatric nursing workforce in China is facing practical problems such as staff shortage, a relatively high proportion of young nurses, and an immature specialized training system, which further exacerbate the difficulty of role adaptation (6). Due to the lack of effective role adjustment strategies, some nurses are prone to role ambiguity, decreased job satisfaction, and even turnover intentions under the pressure of multiple roles. This not only affects the stability of the pediatric nursing team but also restricts the sustainable development of the pediatric nursing discipline (7). Therefore, systematically exploring the current status and characteristics of role conflict and role adaptation among clinical pediatric nurses, and clarifying their core influencing factors, has become an urgent task to meet clinical nursing needs and improve the quality of pediatric nursing. Previous studies (8, 9) have primarily focused on the general prevalence of role conflict and its association with job burnout; however, there remains a lack of in-depth exploration into its specific mechanisms of action within the pediatric nurse population—particularly the mediating pathway of role cognition—and intervenable protective factors, such as the moderating role of psychological support training.

Based on the above clinical realities and disciplinary imperatives, this study aimed to evaluate the current status of role conflict and role adaptation among pediatric clinical nurses, and to examine the intrinsic relationship between these two constructs along with their influencing factors. To clearly delineate the hypothesized relationships, a conceptual framework was developed integrating the mediating pathway of “role conflict → role cognition → role adaptation” and the moderating role of psychological support training. The findings are intended to provide empirical evidence for developing targeted nursing interventions, ultimately helping to alleviate role conflict, enhance role adaptation, reduce job burnout, and promote the stability of the pediatric nursing workforce.

## Methods

### Ethical considerations

This study strictly followed the ethical guidelines of the latest Declaration of Helsinki and China’s medical research ethical review standards. The study protocol was approved by the Medical Ethics Committee of our hospital (Ethics Approval No.: 202601040-1), with all procedures complying with human medical research ethical requirements. Prior to study initiation, uniformly trained researchers provided each participant with written information detailing the research purpose, core content, data collection methods, usage scope, and confidentiality measures. The principle of voluntary participation was clearly communicated, and participants were informed of their right to withdraw unconditionally at any stage without adverse effects on their clinical work, career development, or medical service entitlements. All participants signed a written informed consent form after fully understanding the study details.

### Study design

A cross-sectional survey design with a single-center stratified sampling method was adopted in this study to ensure the representativeness of the sample and the generalizability of the research results.

### Sample size calculation

The core analytical methods of this study included multiple linear regression analysis, mediating effect test, and moderating effect test. Referring to relevant statistical guidelines (10, 11), the sample size for multiple linear regression analysis needs to meet the standard of “number of independent variables × 5~10”. A total of 10 core independent variables (gender, age, department, etc.) were included in this study, and the minimum required sample size was estimated to be 50~100 cases. Combined with the two core scales used in the study (15 items of the Nurse Role Conflict Scale + 25 items of the Nurse Role Adaptation Scale), the sample size was estimated to be no less than 200 cases according to the standard of “total number of scale items × 5”. Meanwhile, considering potential issues such as incomplete questionnaire filling, logical errors, and loss to follow-up during the survey, a 20% redundant sample size was reserved, and the final required sample size was determined to be no less than 240 cases.

### Study population

The inclusion criteria for this study were as follows: (1) holding a valid nurse practicing certificate issued by the National Health Commission of the People’s Republic of China; (2) engaging in direct nursing services in the pediatric clinical frontline for ≥3 months and remaining on the job throughout the study period; (3) possessing good Chinese reading and writing abilities to independently understand the questionnaire content and complete the filling; (4) voluntarily participating in the study and having signed a written informed consent form; (5) not being in a period of long-term leave (e.g., maternity leave, sick leave), post adjustment, or resignation transition during the study period. The exclusion criteria were: (1) non-pediatric clinical frontline nurses, including administrative staff, logistics support personnel, full-time researchers, and teaching staff; (2) advanced practice nurses, student nurses, nurses in standardized training, and short-term temporary nurses; (3) individuals with clearly diagnosed severe mental illnesses (e.g., clinically confirmed depression, anxiety disorder) who were unable to complete the questionnaire normally; (4) questionnaires with incomplete filling (missing ≥3 core items), obvious logical contradictions (e.g., selecting the same option for all items in a single scale), or malicious filling behaviors.

### Survey instruments

#### Basic information questionnaire

Self-designed based on the research purpose and literature review, this questionnaire covered 10 core variable dimensions, specifically including: (1) demographic characteristics: gender, age, marital status, number of children; (2) occupation-related characteristics: department, work experience, professional title, educational background; (3) economic and training-related factors: average monthly income (including basic salary, performance bonuses, etc.), and whether having received psychological support-related training in the past 3 months (e.g., special training on stress management, emotional regulation, psychological counseling skills, job burnout intervention, etc.).

#### Nurse role conflict scale

The full-version scale specifically adapted for pediatric nurses, which was sinicized, revised, and validated by previous scholars (12), was adopted. Derived from the role stress theory framework proposed by Rizzo et al., this scale was localized and adapted to be applicable to Chinese pediatric nursing populations. The scale consisted of 15 items, divided into two core dimensions: (1) Intrarole Conflict (8 items): mainly measuring conflicts caused by internal factors during nurses’ performance of job responsibilities, such as ambiguous responsibility boundaries, inconsistent work standards, conflicting instructions, and mismatch between abilities and job requirements; (2) Interrole Conflict (7 items): mainly measuring conflicts faced by nurses in different role relationships, including work-family conflict, physician-nurse role conflict, nurse-patient (caregiver) expectation conflict, inter-departmental collaboration role conflict, and conflict between social expectations and professional reality. The scale used a 5-point Likert scoring method, with scores ranging from 1 (strongly disagree) to 5 (strongly agree), and the total score ranging from 15 to 75. A higher score indicated a more severe level of role conflict faced by nurses (13). A pre-survey (n=50) was conducted before the formal investigation to test the reliability and validity of the scale. The results showed that the Cronbach’s α coefficient of the total scale was 0.863, the Cronbach’s α coefficient of the Intrarole Conflict dimension was 0.821, and the Cronbach’s α coefficient of the Interrole Conflict dimension was 0.835; the Content Validity Index (CVI) was 0.89 (with item-level CVI all ≥0.80); the construct validity was verified by exploratory factor analysis, with a KMO value of 0.856, Bartlett’s test of sphericity χ²=2345.67 (P<0.001), and 2 common factors extracted, accounting for a cumulative variance explanation rate of 68.72%. Confirmatory factor analysis showed that the model fit well (χ²/df=2.37, RMSEA = 0.058, CFI = 0.92, TLI = 0.91), indicating that the reliability and validity of the scale met the psychometric requirements.

#### Nurse role adaptation scale

A three-dimensional scale constructed based on role adaptation theory and the characteristics of pediatric nursing practice was adopted. After revision by relevant nursing experts, this scale has been validated to have good applicability in the pediatric nurse population (14). The scale consisted of 25 items, divided into three core dimensions: (1) Role Cognition (8 items): mainly measuring nurses’ cognitive level of the responsibility boundaries, core values, team positioning, professional standards, and relevant laws and regulations of their professional roles; (2) Role Behavior (9 items): focusing on the specific behavioral performance of nurses in fulfilling their role responsibilities in clinical practice, including nursing operation skills, disease observation ability, communication and collaboration ability, emergency response ability, and standardized documentation; (3) Role Emotion (8 items): focusing on measuring nurses’ emotional experiences of their professional roles, covering professional identity, job satisfaction, sense of achievement, stress coping ability, and career development willingness. The scale used a 5-point Likert scoring method, with scores ranging from 1 (strongly disagree) to 5 (strongly agree), and the total score ranging from 25 to 125. A higher score indicated a better level of role adaptation among nurses (15). The reliability and validity of the scale were tested during the pre-survey phase (n=50), and the results showed that the Cronbach’s α coefficient of the total scale was 0.887, and the CVI coefficients of the Role Cognition, Role Behavior, and Role Emotion dimensions were 0.832, 0.846, and 0.859, respectively; the Content Validity Index (CVI) was 0.91 (with item-level CVI all ≥0.85); in terms of construct validity, the KMO value was 0.872, Bartlett’s test of sphericity χ²=3126.89 (P<0.001), and 3 common factors were extracted by exploratory factor analysis, accounting for a cumulative variance explanation rate of 71.35%. Confirmatory factor analysis showed that the three-dimensional model had good fit indices (χ²/df=2.15, RMSEA = 0.052, CFI = 0.94, TLI = 0.93), which met the psychometric standards and could be used for data collection in this study.

### Survey process

The study was conducted from June 2025 to September 2025 in a tertiary grade A specialized children’s hospital in China, adopting a mixed survey method combining online and offline approaches to adapt to the work scenarios of different hospitals and the work rhythms of nurses. Prior to the survey, all researchers involved in data collection received unified training, covering research background, questionnaire filling standards, informed consent signing procedures, communication skills, and privacy protection requirements. Only those who passed the assessment were allowed to participate in the survey. For offline surveys, researchers carried paper questionnaires to the pediatric departments of each hospital. With the assistance of department head nurses, questionnaires were distributed centrally during relatively free periods of nurses’ work (e.g., lunch breaks, before work). The research purpose and filling precautions were explained on-site, and participants were guided to complete the questionnaires independently and return them on the spot. For online surveys, exclusive anonymous questionnaire links were generated through the Wenjuanxing platform, which were forwarded to nurses’ work groups with the assistance of department head nurses, accompanied by detailed filling instructions, reminding nurses to complete and submit online within one week. After questionnaire collection, two researchers independently conducted data verification and entry, using a double-entry method for cross-checking to ensure the accuracy of data entry. For questionnaires with missing ≤2 items, multiple imputation was used for data supplementation; for questionnaires with missing >2 items, obvious logical errors, or all items selected as the same option, they were judged as invalid and excluded. All data were verified to be correct before being imported into statistical software for subsequent analysis, with strict quality control throughout the process.

### Statistical analysis

Data processing and statistical analyses were performed using SPSS 26.0 and AMOS 24.0. All statistical tests were two-tailed, with a significance level set at α = 0.05. Descriptive statistics were expressed as mean ± standard deviation (x¯ ± s) for continuous variables and as frequencies (n) and percentages (%) for categorical variables. Intergroup comparisons were conducted using independent samples t−tests for two-group comparisons, one−way analysis of variance (ANOVA) for comparisons involving more than two groups, and chi−square tests for categorical variables.

Pearson correlation analysis was used to examine the strength and direction of associations between the dimensions and total scores of role conflict and role adaptation. For multivariate analysis, stepwise multiple linear regression (entry criterion α = 0.05, removal criterion α = 0.10) was employed to identify independent factors associated with role conflict and role adaptation. To account for the clinical heterogeneity across departments, the department variable was coded as a continuous measure based on a predefined clinical stress level derived from a preliminary survey and expert panel consensus (1 = outpatient, 2 = other pediatric-related departments, 3 = pediatric operating room, 4 = pediatric surgery, 5 = pediatric internal medicine, 6 = neonatology, 7 = pediatric emergency department, 8 = pediatric ICU), allowing for the examination of a dose−response relationship. A sensitivity analysis treating department as a categorical variable using dummy coding was conducted to verify the robustness of the findings (**Supplementary Table 1**). Multicollinearity was assessed using the variance inflation factor (VIF), with VIF < 10 indicating no significant multicollinearity. Control variables were selected based on theoretical considerations and prior empirical evidence linking demographic and occupational characteristics to role stress and adaptation in nursing populations, including gender, age, work experience, professional title, marital status, number of children, and psychological support training.

For mechanism testing, Model 4 of the Hayes PROCESS macro was used to test the mediating effect of role cognition on the relationship between role conflict and role adaptation, and Model 1 was used to test the moderating effect of psychological support training. Both models estimated 95% confidence intervals (CIs) using 5000 bootstrap samples; an effect was considered statistically significant if the CI did not contain zero. Bonferroni correction was applied for multiple pairwise comparisons to control for Type I error. All statistical procedures adhered to corresponding methodological standards to ensure the scientific rigor and accuracy of the results.

## Results

### Demographic characteristics of participants and descriptive statistics of core variables

A total of 426 pediatric clinical nurses were included in this study, with a predominance of female participants (94.13%) and a relatively high proportion of nurses aged 26–35 years (55.87%) and with 4–10 years of work experience (43.43%). In terms of department distribution, the sample covered 8 pediatric subspecialties, including pediatric internal medicine (28.87%), pediatric surgery (22.30%), and pediatric ICU (12.68%), among others. The majority of participants held a bachelor’s degree (91.08%) and had an average monthly income of 5001–8000 RMB (69.25%), with 37.09% having received psychological support training in the past 3 months (**Table 1**).

**Table 1:** Demographic characteristics of participants and descriptive statistics of role conflict and role adaptation scores (n=426).

Variable​	Category/group​	Number (n)​	Percentage (%)​	Total role conflict score ( ± s)​	Total role adaptation score ( ± s)​	Test statistic (t/χ²/F)​	P
Gender​ ​	Male​	25​	5.87	55.48 ± 6.91​	81.05 ± 11.37​	t=2.763​	0.006​
	Female​	401​	94.13	52.03 ± 7.62​	85.68 ± 10.29​	​	​
Age​ ​ ​	≤25 years​	112​	26.29​	56.52 ± 7.03​	80.37 ± 10.76​	F=11.845​	<0.001​
	26–35 years​	238​	55.87​	52.18 ± 7.36​	86.14 ± 10.05​	​	​
	≥36 years​	76​	17.84​	48.93 ± 7.95​	89.42 ± 9.28​	​	​
Department​ ​ ​ ​ ​ ​ ​ ​	Neonatology​	46​	10.80​	57.19 ± 6.85​	79.64 ± 11.12​	F=16.327​	<0.001​
	Pediatric internal medicine​	123​	28.87​	53.02 ± 7.29​	85.07 ± 10.21​	​	​
	Pediatric surgery​	95​	22.30​	51.76 ± 7.68​	86.43 ± 9.81​	​	​
	Pediatric operating room​	42​	9.86​	50.58 ± 8.07​	87.65 ± 9.59​	​	​
	Pediatric emergency department​	30​	7.04​	58.47 ± 6.63​	78.19 ± 11.62​	​	​
	Pediatric outpatient department​	24​	5.63​	48.82 ± 7.98​	89.53 ± 8.89​	​	​
	Pediatric ICU​	54​	12.68​	59.23 ± 6.47​	77.85 ± 11.73​	​	​
	Other pediatric related departments​	12​	2.82​	49.25 ± 8.21​	88.86 ± 9.12​	​	​
Work experience​ ​ ​	≤3 years​	158​	37.09​	56.27 ± 6.98​	82.15 ± 10.48​	F=10.362​	<0.001​
	4–10 years​	185​	43.43​	51.89 ± 7.42​	86.37 ± 9.93​	​	​
	≥11 years​	83​	19.48​	47.95 ± 8.01​	89.76 ± 8.75​	​	​
Professional title​ ​ ​ ​	Nurse​	152​	35.68​	56.58 ± 6.99​	82.27 ± 10.61​	F=8.743​	<0.001​
	Senior nurse​	189​	44.37​	52.71 ± 7.35​	85.98 ± 10.01​	​	​
	Nurse practitioner​	75​	17.61​	49.23 ± 7.88​	88.85 ± 9.21​	​	​
	Chief nurse and above​	10​	2.35​	47.06 ± 8.24​	91.52 ± 7.98​	​	​
Educational background​ ​	Technical secondary school​	38​	8.92​	53.26 ± 7.89​	83.18 ± 11.21​	t=1.135​	0.257​
	Bachelor’s degree​	388​	91.08​	52.21 ± 7.56​	85.49 ± 10.33​	​	​
Marital status​ ​ ​	Unmarried​	138​	32.40​	52.89 ± 7.79​	83.02 ± 10.87​	t=2.368​	0.018​
	Married​	272​	63.85​	52.07 ± 7.45​	86.59 ± 10.12​	​	​
	Divorced/widowed​	16​	3.76​	54.67 ± 8.05​	82.59 ± 11.43​	​	​
Number of children​ ​ ​	0​	182​	42.72​	50.71 ± 7.89​	85.86 ± 10.17​	F=7.128​	0.001​
	1​	173​	40.61​	53.04 ± 7.35​	85.02 ± 10.51​	​	​
	≥2​	71​	16.67​	56.48 ± 6.98​	83.65 ± 10.92​	​	​
Average monthly income​ ​ ​ ​	≤5000 RMB​	68​	15.96​	53.12 ± 7.71​	84.15 ± 10.65​	F=1.032​	0.378​
	5001–8000 RMB​	295​	69.25​	52.38 ± 7.53​	85.42 ± 10.31​	​	​
	8001–12000 RMB​	51​	11.97​	52.07 ± 7.68​	85.79 ± 10.18​	​	​
	≥12001 RMB​	12​	2.82​	51.89 ± 8.05​	86.83 ± 9.82​	​	​
Psychological support training in recent 3 months​ ​	Yes​	158​	37.09​	49.23 ± 7.29​	89.78 ± 9.21​	t=7.965​	<0.001​
	No​	268​	62.91​	54.76 ± 7.36​	82.35 ± 10.61​	​	​

The total score of role conflict among pediatric nurses was 52.46 ± 7.55 (range: 15–75), and the total score of role adaptation was 85.20 ± 10.48 (range: 25–125). Significant differences in role conflict and role adaptation scores were observed across different demographic and occupational subgroups (**Table 1**). Specifically, male nurses, younger nurses (≤25 years), those working in high-pressure departments (pediatric ICU, emergency department, neonatology), nurses with shorter work experience (≤3 years), and those with lower professional titles (staff nurse) reported higher role conflict scores. Conversely, nurses who were married, had more children, received psychological support training, or worked in outpatient departments or other non-acute care settings exhibited better role adaptation. No significant differences were found in role conflict or adaptation scores based on educational background or average monthly income (P>0.05).

### Correlation between role conflict and role adaptation

Pearson correlation analysis revealed significant correlations among role conflict, role adaptation, and their respective dimensions (**Table 2**). Role conflict (including intrarole and interrole conflict) was significantly negatively correlated with total role adaptation and its three dimensions (role cognition, role behavior, role emotion), with correlation coefficients ranging from -0.539 to -0.728 (all P<0.001). Among these, role emotion showed the strongest negative correlation with role conflict, while role cognition exhibited the highest positive correlation with total role adaptation (r=0.843, P<0.001). Additionally, intrarole conflict and interrole conflict were strongly positively correlated (r=0.779, P<0.001), and the three dimensions of role adaptation were mutually positively correlated, with correlation coefficients between 0.765 and 0.894 (all P<0.001).

**Table 2:** ​Pearson correlation analysis between role conflict, role adaptation dimensions and total scores (n=426)​.

Variable​	1. Intrarole conflict​	2. Interrole conflict​	3. Total role conflict score​	4. Role cognition​	5. Role behavior​	6. Role emotion​	7. Total role adaptation score​
1. Intrarole conflict​	1​	​	​	​	​	​	​
2. Interrole conflict​	r=0.779P<0.001​	1​	​	​	​	​	​
3. Total role conflict score​	r=0.892P<0.001​	r=0.868P<0.001​	1​	​	​	​	​
4. Role cognition​	r=-0.631P<0.001​	r=-0.583P<0.001​	r=-0.674P<0.001​	1​	​	​	​
5. Role behavior​	r=-0.588P<0.001​	r=-0.539P<0.001​	r=-0.637P<0.001​	r=0.765P<0.001​	1​	​	​
6. Role emotion​	r=-0.654P<0.001​	r=-0.608P<0.001​	r=-0.699P<0.001​	r=0.792P<0.001​	r=0.809P<0.001​	1​	​
7. Total role adaptation score​	r=-0.683P<0.001​	r=-0.631P<0.001​	r=-0.728P<0.001​	r=0.843P<0.001​	r=0.860P<0.001​	r=0.894P<0.001​	1​

### Influencing factors of role conflict among pediatric nurses

Multiple linear regression analysis identified seven independent influencing factors of role conflict, including gender, age, department, work experience, professional title, number of children, and psychological support training (**Table 3**). The model explained 46.7% of the total variance in role conflict (adjusted R²=0.467, F = 33.965, P<0.001). Male gender (β=0.121, P = 0.006), younger age (β=-0.281, P<0.001), working in high-pressure departments (e.g., pediatric ICU, emergency department; β=0.221, P<0.001), shorter work experience (β=-0.251, P<0.001), lower professional title (β=-0.192, P<0.001), and more children (β=0.148, P = 0.002) were associated with higher role conflict. In contrast, receiving psychological support training was a protective factor against role conflict (β=-0.235, P<0.001). No multicollinearity was detected among variables (VIF<2).

**Table 3:** ​Multiple linear regression analysis of factors influencing role conflict among pediatric nurses.

Independent variable​	Coding method​	B​	SE​	β​	t-value​	P​	95%CI​	VIF​
Gender (reference: female)​	Male=1; female=0​	2.793​	1.012​	0.121​	2.760​	0.006​	0.807-4.779​	1.048​
Age (reference: ≤25 years)​	26–35 years=1; ≥36 years=2​	-2.587​	0.495​	-0.281​	-5.226​	<0.001​	-3.561-1.613​	1.312​
Department (reference: outpatient)​	Neonatology=1; internal medicine=2; surgery=3; operating room=4; emergency=5; ICU = 6; others=7​	3.264​	0.673​	0.221​	4.849​	<0.001​	1.942-4.586​	1.476​
Work experience (reference: ≤3 years)​	4–10 years=1; ≥11 years=2​	-2.435​	0.528​	-0.251​	-4.612​	<0.001​	-3.473-1.397​	1.354​
Professional title (reference: nurse)​	Senior nurse=1; nurse practitioner=2; chief nurse and above=3​	-2.096​	0.549​	-0.192​	-3.818​	<0.001​	-3.178-1.014​	1.287​
Number of children (reference: 0)​	1 = 1; ≥2 = 2​	1.932​	0.637​	0.148​	3.033​	0.002​	0.681-3.183​	1.179​
Psychological support training (reference: no)​	Yes=1; no=0​	-4.028​	0.796​	-0.235​	-5.060​	<0.001​	-5.594-2.462​	1.083​
Constant​	​	59.537​	2.214​	-​	26.891​	<0.001​	55.185-63.889​	-​

​Stepwise regression analysis was adopted (α-entry=0.05, α-removal=0.10). Model goodness of fit: R²=0.481, adjusted R²=0.467, F = 33.965, P<0.001. Variance inflation factor (VIF) was all <2, indicating no multicollinearity.​ Department was coded based on a predefined clinical stress level derived from a preliminary survey and expert panel consensus, with higher scores indicating higher perceived clinical stress (outpatient as reference; coded values for other departments reflect increasing stress levels). The positive β value indicates that as departmental stress level increases, role conflict significantly increases.

### Influencing factors of role adaptation among pediatric nurses

Five independent factors influencing role adaptation were identified through multiple linear regression analysis: gender, age, department, marital status, and psychological support training (**Table 4**). The model accounted for 41.3% of the variance in role adaptation (adjusted R²=0.413, F = 29.987, P<0.001). Female gender (β=-0.115, P = 0.004), older age (β=0.208, P<0.001), married status (β=0.108, P = 0.009), and receiving psychological support training (β=0.230, P<0.001) were associated with better role adaptation. Working in high-pressure departments (e.g., pediatric ICU, emergency department) was a risk factor for poor role adaptation (β=-0.172, P<0.001).

**Table 4:** Multiple linear regression analysis of factors influencing role adaptation among pediatric nurses.

Independent variable​	Coding method​	B​	SE​	β​	t​	P	95%CI​	VIF​
Gender (reference: female)​	Male=1; female=0​	-3.687​	1.263​	-0.115​	-2.920​	0.004​	-6.174-1.199​	1.045​
Age (reference: ≤25 years)​	26–35 years=1; ≥36 years=2​	2.543​	0.607​	0.208​	4.189​	<0.001​	1.349-3.737​	1.308​
Department (reference: outpatient)​	Neonatology=1; internal medicine=2; surgery=3; operating room=4; emergency=5; ICU = 6; others=7​	-2.974​	0.823​	-0.172​	-3.614​	<0.001​	-4.592-1.356​	1.469​
Marital status (reference: unmarried)​	Married=1; divorced/widowed=2​	2.712​	1.041​	0.108​	2.605​	0.009​	0.665-4.759​	1.118​
Psychological support training (reference: no)​	Yes=1; no=0​	4.956​	0.982​	0.230​	5.047​	<0.001​	3.027-6.885​	1.079​
Constant​	​	78.015​	2.732​	-​	28.556​	<0.001​	72.641-83.389​	-​

Stepwise regression analysis was adopted (α-entry=0.05, α-removal=0.10). Model goodness of fit: R²=0.427, adjusted R²=0.413, F = 29.987, P<0.001. Variance inflation factor (VIF) was all <2, indicating no multicollinearity.​ Department was coded based on a predefined clinical stress level derived from a preliminary survey and expert panel consensus, with higher scores indicating higher perceived clinical stress (outpatient as reference; coded values for other departments reflect increasing stress levels). The negative β value indicates that as departmental stress level increases, role adaptation significantly decreases.

### Moderating effect of psychological support training

Hierarchical regression analysis confirmed a significant moderating effect of psychological support training on the relationship between role conflict and role adaptation (**Table 5**). After adding the interaction term (role conflict × psychological support training) to the model, the explained variance increased by 3.8% (ΔR²=0.038, F = 18.426, P<0.001). The interaction term was statistically significant (β=0.153, P<0.001), indicating that psychological support training weakened the negative impact of role conflict on role adaptation. Specifically, for nurses who received psychological support training, the negative association between role conflict and role adaptation was less pronounced compared to those who did not.

**Table 5:** Hierarchical regression analysis of the moderating effect of psychological support training on the relationship between role conflict and role adaptation.

Model	Independent variable	B	SE	β	t	P	R²	ΔR²	F(ΔR²)	P(ΔR²)
1	Control variables (gender, age, department, marital status)	-	-	-	-	-	0.183	-	-	-
	Total role conflict score (X)	-0.581	0.066	-0.420	-8.803	<0.001				
2	Variables in model 1 + psychological support training (M)	-	-	-	-	-	0.259	0.076	36.248	<0.001
	Total role conflict score (X)	-0.606	0.063	-0.438	-9.619	<0.001				
	Psychological support training (M)	4.312	0.719	0.201	5.997	<0.001				
3	Variables in model 2 + X×M (interaction term)	-	-	-	-	-	0.297	0.038	18.426	<0.001
	Total role conflict score (X)	-0.778	0.085	-0.563	-9.153	<0.001				
	Psychological support training (M)	5.203	0.857	0.242	6.071	<0.001				
	Role conflict × psychological support training (interaction term)	0.193	0.046	0.153	4.221	<0.001				

Hierarchical multiple regression analysis was used. The moderating effect was significant (interaction term P<0.001), and simple slope analysis was performed subsequently to draw the moderation effect diagram.

### Mediating effect of role cognition

Bootstrap analysis (5000 samples) demonstrated a significant mediating effect of role cognition on the relationship between role conflict and role adaptation (**Table 6**). The total effect of role conflict on role adaptation was -0.728 (95%CI: -0.845 to -0.611, P<0.001), with a direct effect of -0.321 (95%CI: -0.450 to -0.192, P<0.001) and an indirect effect of -0.407 (95%CI: -0.503 to -0.312, P<0.001). Role cognition accounted for 55.91% of the total effect, indicating that role conflict negatively affects role adaptation both directly and indirectly by impairing nurses’ role cognition.

**Table 6:** Bootstrap analysis of the mediating effect of role cognition on the relationship between role conflict and role adaptation​.

Path​	Effect size​	SE​	95%CI (bootstrap)​	Z​	P​	Mediating effect ratio (%)​
Total effect: role conflict → role adaptation​	-0.728​	0.060​	[-0.845, -0.611]​	-12.133​	<0.001​	-​
Direct effect: role conflict → role adaptation​	-0.321​	0.066​	[-0.450, -0.192]​	-4.864​	<0.001​	-​
Indirect effect: role conflict → role cognition → role adaptation​	-0.407​	0.049​	[-0.503, -0.312]​	-8.306​	<0.001​	55.91​

​Hayes PROCESS macro (model 4) was used with 5000 bootstrap samples. The 95%CI did not contain 0, indicating a significant mediating effect.​

### Differences in role conflict and adaptation across departments

One-way ANOVA with Bonferroni *post-hoc* test showed significant differences in role conflict and role adaptation scores among nurses from different pediatric departments (**Table 7**). Pediatric ICU nurses had the highest role conflict score (59.23 ± 6.47) and the lowest role adaptation score (77.85 ± 11.73), followed by emergency department and neonatology nurses. In contrast, outpatient department nurses exhibited the lowest role conflict (48.82 ± 7.98) and the highest role adaptation (89.53 ± 8.89). Pairwise comparisons revealed that role conflict scores in pediatric ICU, emergency department, and neonatology were significantly higher than those in outpatient, surgical, and operating room departments (all P<0.001), while role adaptation scores were significantly lower (all P<0.001). No significant differences were observed between pediatric ICU and emergency department nurses (P>0.05) or between neonatology and emergency department nurses (P>0.05).

**Table 7:** *Post-hoc* test analysis of role conflict and adaptation among nurses in different pediatric departments​.

Department​	Number (n)​	Total role conflict score ( ± s)​	Total role adaptation score ( ± s)​	*Post-hoc* P-value (vs. neonatology)​	*Post-hoc* P-value (vs. ICU)​
Neonatology​	46​	57.19 ± 6.85​	79.64 ± 11.12​	-​	0.432​
Pediatric ICU​	54​	59.23 ± 6.47​	77.85 ± 11.73​	0.432​	-​
Pediatric emergency department​	30​	58.47 ± 6.63​	78.19 ± 11.62​	0.568​	0.893​
Pediatric internal medicine​	123​	53.02 ± 7.29​	85.07 ± 10.21​	0.003​	<0.001​
Pediatric surgery​	95​	51.76 ± 7.68​	86.43 ± 9.81​	<0.001​	<0.001​
Pediatric operating room​	42​	50.58 ± 8.07​	87.65 ± 9.59​	<0.001​	<0.001​
Pediatric outpatient department​	24​	48.82 ± 7.98​	89.53 ± 8.89​	<0.001​	<0.001​
Other pediatric related departments​	12​	49.25 ± 8.21​	88.86 ± 9.12​	<0.001​	<0.001​

​One-way ANOVA + Bonferroni *post-hoc* test (controlling type I error); F (role conflict) =16.327, P<0.001; F (role adaptation) =13.154, P<0.001.

## Discussion

### Current status of role conflict and role adaptation among pediatric nurses

This study identified a moderate level of role conflict and relatively favorable role adaptation among pediatric clinical nurses. Such role conflict is a common phenomenon in specialized nursing fields, where the confluence of high work intensity and multifaceted role expectations inherently generates psychological strain. Pediatric nursing is distinguished by unique clinical complexities, including the rapid progression of childhood illnesses, limited communication capacities of young patients, and heightened emotional and care demands from families (16, 17). Consistent with contemporary perspectives on emotional labor in pediatric nursing (18, 19), these factors require nurses to manage not only clinical tasks but also their own emotional expressions and responses, thereby intensifying the psychological demands of the role. These factors require nurses to simultaneously fulfill caregiving, educational, and supportive roles, creating an inherent risk of role conflict (20). In contrast, the observed positive role adaptation may stem from the professional identity cultivated through long-term clinical practice and the supportive frameworks provided by organizational management systems (21). This aligns with the understanding that role adaptation is a dynamic process shaped by the interaction of individual professional growth and institutional support mechanisms (22, 23).

### Demographic and occupational factors influencing role conflict

Multiple regression analysis highlighted several key determinants of role conflict among pediatric nurses. Male nurses reported higher levels of role conflict, a finding potentially linked to the gender imbalance in the nursing profession and the unique social and professional pressures faced by men in a female-dominated field. Younger nurses and those with shorter work experience also exhibited elevated role conflict, which is consistent with the professional development trajectory of novice nurses—individuals in this stage often grapple with role ambiguity and insufficient clinical competence when adapting to the rigorous demands of pediatric care (24). Additionally, nurses in high-acuity departments (pediatric ICU, emergency department, neonatology) experienced significantly higher role conflict compared to their counterparts in outpatient or surgical settings (25). This underscores the profound impact of clinical environment characteristics—such as high patient severity, frequent emergency situations, and intensive care requirements—on exacerbating tensions between role expectations and available resources (26, 27). Nurses with more children also faced greater role conflict, highlighting the pervasive challenge of work-family balance in pediatric nursing, where emotional engagement with sick children must be reconciled with personal family responsibilities (28). Nurses with more children also faced greater role conflict (29), highlighting the pervasive challenge of work-family balance in pediatric nursing, where emotional engagement with sick children must be reconciled with personal family responsibilities. Notably, while having more children was associated with higher role conflict, being married was paradoxically associated with better role adaptation. This seemingly contradictory finding may be explained through the lens of the work-family enrichment model and the social support buffering hypothesis: the quality of spousal emotional and practical support, rather than marital status per se, likely serves as a critical resource that helps nurses navigate work-related stress and maintain role adaptation (30).

### Factors influencing role adaptation and its correlation with role conflict

Role adaptation among pediatric nurses was positively associated with female gender, older age, marital status, and participation in psychological support training, while being negatively correlated with employment in high-pressure departments. Female nurses may possess inherent advantages in emotional empathy and communication skills, which are particularly valuable in pediatric care that emphasizes family-centered approaches. Married nurses often demonstrate greater life stability and coping resources, enabling them to navigate work-related stress more effectively. The protective effect of psychological support training on role adaptation is noteworthy, as targeted psychological interventions have been shown to enhance emotional resilience and role adjustment capabilities (31). Pearson correlation analysis further revealed a strong negative association between role conflict and role adaptation, emphasizing that mitigating role conflict is a critical prerequisite for improving role adaptation. This correlation is grounded in role theory, which posits that incompatible role expectations (conflict) impede the effective performance of role responsibilities (adaptation) (32).

### Mediating effect of role cognition

Bootstrap analysis confirmed that role cognition plays a significant mediating role in the relationship between role conflict and role adaptation, accounting for over half of the total effect. This finding indicates that role conflict exerts dual negative impacts on role adaptation: a direct effect and an indirect effect operating through impaired role cognition. Specifically, high role conflict can lead to unclear role boundaries, ambiguous professional values, and inadequate understanding of role expectations, all of which undermine effective role performance (33, 34). For instance, nurses experiencing intense role conflict may struggle to define their core responsibilities, resulting in ineffective role behaviors and reduced emotional engagement with their profession. This mediating mechanism can be further understood through Social Cognitive Theory and Role Identity Theory (35, 36): role conflict may disrupt nurses’ self-concept and outcome expectations, thereby weakening their professional identity and role-related self-efficacy, ultimately impairing role adaptation. The findings underscore the need to explicitly recognize and support the emotional labor inherent in pediatric nursing (19, 37). Beyond generic psychological support training, healthcare institutions should implement structured programs that teach specific emotion regulation strategies—such as cognitive reappraisal and boundary management—and create a workplace culture where the emotional challenges of caring for seriously ill children and distressed families are openly acknowledged and discussed. Such organizational-level interventions are essential for sustaining nurses’ psychological well-being and enhancing their capacity for role adaptation.

### Moderating effect of psychological support training

Psychological support training was found to moderate the relationship between role conflict and role adaptation, exerting a buffering effect that weakens the negative impact of role conflict. This aligns with Conservation of Resources (COR) theory, which suggests that psychological resources—such as stress management skills and emotional regulation strategies—can offset the depletion of resources caused by role conflict (38, 39). Moreover, from the perspective of the Job Demands-Resources (JD-R) Model (40), psychological support training may function as a job resource that balances the high demands inherent in pediatric nursing, thereby reducing burnout and enhancing engagement. Nurses who participated in psychological support training are better equipped to cope with role-related stress, reducing the risk of emotional exhaustion and enhancing their ability to adapt to multifaceted role demands. Importantly, psychological support training may also influence the mediating pathway by enhancing role cognition—improving role clarity and self-efficacy—thereby indirectly promoting role adaptation. This interplay between moderating and mediating mechanisms suggests that psychological support training operates at multiple levels to protect nurses’ well-being. This finding underscores the practical value of psychological support programs in pediatric nursing management, as investing in such training can foster resilience even in high-conflict environments, ultimately improving professional well-being and care quality (41). Notably, the relatively low proportion of nurses who had received psychological support training in this study indicates a critical gap in current organizational support systems that warrants attention.

### Interdepartmental differences in role conflict and adaptation

Significant interdepartmental variations in role conflict and adaptation were observed, with pediatric ICU nurses reporting the highest conflict and lowest adaptation, followed by emergency and neonatology nurses. These departments are characterized by high patient acuity, complex care needs, and frequent ethical dilemmas, which intensify role conflict by amplifying mismatches between role expectations and available resources (42). In contrast, outpatient department nurses exhibited the lowest role conflict and highest adaptation, as outpatient settings typically involve more predictable care routines, lower patient acuity, and reduced role overload (43). These interdepartmental differences provide targeted insights for nursing management, emphasizing the need for tailored interventions rather than a one-size-fits-all approach (44, 45). For example, high-conflict departments may benefit from optimized staffing ratios, flexible scheduling, and peer support networks, while general pediatric departments can maintain supportive practices and facilitate knowledge sharing with high-pressure units to promote collective improvement (46).

### Clinical nursing and management implications

Based on the study findings, targeted strategies to alleviate role conflict, improve role adaptation, and enhance the stability of the pediatric nursing workforce should be implemented. Healthcare institutions should prioritize role clarification by developing department-specific guidelines that define core responsibilities, particularly in high-pressure settings such as pediatric ICU and emergency departments, while providing tailored training programs for novice nurses focusing on clinical skills, child and family communication, and role transition to reduce role ambiguity (47). Expanding psychological support systems is critical, including offering regular workshops on stress management, emotional regulation, and work-family balance, as well as establishing confidential professional counseling services to support nurses experiencing high role conflict or burnout (48, 49). Department-specific management strategies should be adopted, such as adjusting staffing ratios and implementing flexible scheduling in high-conflict departments to reduce workload and facilitate work-family balance, while maintaining and promoting supportive practices in outpatient and surgical departments. Enhancing professional development opportunities through specialized certifications and clear career progression pathways can strengthen professional identity and reduce turnover intentions, complemented by recognition programs that acknowledge the unique contributions of pediatric nurses to foster a positive work environment (50, 51). Additionally, targeted support for male nurses—such as mentorship programs to address gender-specific challenges—and flexible work arrangements for nurses with multiple children can help alleviate work-family conflict and improve overall role adaptation, ultimately contributing to enhanced care quality for pediatric patients. From a mental health nursing perspective, these interventions also hold promise for promoting the psychological well-being of pediatric nurses, which is integral to sustaining a resilient and compassionate nursing workforce.

## Limitations

This study has several limitations that should be acknowledged. The cross-sectional design precludes the establishment of causal relationships between variables, and future longitudinal studies are needed to explore the dynamic changes in role conflict, role cognition, and role adaptation over time. The single-center sampling approach may limit the generalizability of findings to other regions or healthcare systems, highlighting the need for multi-center studies with larger sample sizes to validate the results. Data collection relied on self-reported questionnaires, which may be subject to response bias, such as social desirability bias; future research could incorporate objective measures, such as supervisor evaluations or clinical performance indicators, to complement self-reported data. Additionally, the study focused on individual and organizational factors without exploring the impact of broader societal influences, such as social perceptions of nursing or healthcare policies, which warrant further investigation. Additionally, the study focused primarily on individual-level factors without exploring the role of team- or organizational-level variables—such as leadership style, unit safety culture, or interdisciplinary collaboration—within the described mechanisms. The content, duration, and intensity of “psychological support training” were not differentiated, which represents a direction for future, more detailed research. Broader societal influences, such as social perceptions of nursing or healthcare policies, also warrant further investigation. Despite these limitations, the findings provide valuable empirical evidence for understanding the role dynamics of pediatric nurses and inform targeted interventions to improve their professional well-being and the quality of care provided to children.

## Conclusion

In conclusion, this study provides a comprehensive assessment of the current status, influencing factors, and underlying mechanisms of role conflict and role adaptation among pediatric clinical nurses. The findings indicate that pediatric nurses experience a moderate level of role conflict alongside relatively favorable role adaptation, with notable variations across demographic characteristics, occupational factors, and clinical departments. Male gender, younger age, shorter work experience, employment in high-acuity departments (e.g., pediatric ICU, emergency department), and having more children were identified as risk factors for increased role conflict, whereas female gender, older age, married status, and participation in psychological support training emerged as protective factors for better role adaptation. Correlation and regression analyses confirmed a strong negative association between role conflict and role adaptation, with role cognition playing a significant mediating role (accounting for over half of the total effect) and psychological support training exerting a critical moderating effect that buffers the adverse impact of role conflict on adaptation. Interdepartmental differences further revealed that nurses in high-pressure settings experience greater role conflict and poorer adaptation compared with those in outpatient or non-acute care departments. Based on these findings, targeted interventions are warranted, including refining department-specific role guidelines, expanding psychological support systems, optimizing staffing and scheduling in high-conflict departments, enhancing professional development pathways, and providing tailored support for vulnerable groups (e.g., male nurses, nurses with multiple children). Future research may adopt longitudinal designs to examine the dynamic evolution of role conflict and adaptation over time, employ multi-center sampling to enhance generalizability, integrate objective assessment measures to complement self-reported data, and explore the influence of broader societal and policy factors. In addition, future studies could focus on developing and evaluating evidence-based intervention programs tailored to pediatric nursing contexts, with the aim of further mitigating role conflict, strengthening role adaptation, and promoting the long-term stability and professional well-being of the pediatric nursing workforce, ultimately contributing to the improvement of pediatric healthcare quality.

## Data Availability

The original contributions presented in the study are included in the article/**Supplementary Material**. Further inquiries can be directed to the corresponding authors.
