# Supplementary material for: Pediatric clinical nurses’ role conflict and adaptation status: time for improvement
Source: Front Psychiatry. 2026 May 12;17:1754467. doi: 10.3389/fpsyt.2026.1754467 (PMC13201488; doi:10.3389/fpsyt.2026.1754467)
Supplement: Supplementary file 1 [file Table1.docx]

Supplementary Table S1. Sensitivity analysis: Multiple linear regression with department as categorical variable (dummy coding) for role conflict and role adaptation

|  |  | Role Conflict (n = 426) | | | |  | Role Adaptation (n = 426) | | | |  |
| --- | --- | --- | --- | --- | --- | --- | --- | --- | --- | --- | --- |
| Variable | Coding | β (SE) | Standardized β | t | P |  | β (SE) | Standardized β | t | P |  |
| Gender (ref: female) | Male = 1 | 2.76 (1.02) | 0.120 | 2.71 | 0.007 |  | -3.65 (1.27) | -0.113 | -2.87 | 0.004 |  |
| Age (ref: ≤25 years) | 26–35 years = 1; ≥36 years = 2 | -2.59 (0.50) | -0.282 | -5.18 | <0.001 |  | 2.52 (0.61) | 0.206 | 4.13 | <0.001 |  |
| Department (ref: outpatient) |  |  |  |  |  |  |  |  |  |  |  |
|  | Neonatology | 8.35 (1.86) | 0.202 | 4.49 | <0.001 |  | -9.70 (2.28) | -0.168 | -4.25 | <0.001 |  |
|  | Pediatric internal medicine | 4.19 (1.70) | 0.150 | 2.46 | 0.014 |  | -4.42 (2.09) | -0.113 | -2.11 | 0.035 |  |
|  | Pediatric surgery | 2.94 (1.72) | 0.099 | 1.71 | 0.088 |  | -3.01 (2.11) | -0.072 | -1.43 | 0.154 |  |
|  | Pediatric operating room | 1.76 (1.85) | 0.043 | 0.95 | 0.342 |  | -1.88 (2.27) | -0.033 | -0.83 | 0.407 |  |
|  | Pediatric emergency department | 9.67 (1.97) | 0.201 | 4.91 | <0.001 |  | -11.32 (2.42) | -0.169 | -4.68 | <0.001 |  |
|  | Pediatric ICU | 10.41 (1.86) | 0.270 | 5.60 | <0.001 |  | -11.69 (2.28) | -0.218 | -5.13 | <0.001 |  |
|  | Other pediatric related departments | 0.43 (2.39) | 0.006 | 0.18 | 0.857 |  | -0.66 (2.94) | -0.006 | -0.22 | 0.823 |  |
| Work experience (ref: ≤3 years) | 4–10 years = 1; ≥11 years = 2 | -2.41 (0.53) | -0.249 | -4.55 | <0.001 |  | — | — | — | — |  |
| Professional title (ref: nurse) | Senior nurse = 1; nurse practitioner = 2; chief nurse and above = 3 | -2.09 (0.55) | -0.191 | -3.80 | <0.001 |  | — | — | — | — |  |
| Number of children (ref: 0) | 1 = 1; ≥2 = 2 | 1.91 (0.64) | 0.146 | 2.98 | 0.003 |  | — | — | — | — |  |
| Marital status (ref: unmarried) | Married = 1; divorced/widowed = 2 | — | — | — | — |  | 2.68 (1.05) | 0.107 | 2.55 | 0.011 |  |
| Psychological support training (ref: no) | Yes = 1 | -4.00 (0.80) | -0.233 | -5.00 | <0.001 |  | 4.92 (0.98) | 0.228 | 5.02 | <0.001 |  |
| Constant |  | 59.12 (2.23) |  | 26.51 | <0.001 |  | 78.21 (2.74) |  | 28.54 | <0.001 |  |
| Model fit |  | R² = 0.483, adjusted R² = 0.461, F = 22.16, P < 0.001 |  |  |  |  | R² = 0.432, adjusted R² = 0.410, F = 21.08, P < 0.001 |  |  |  |  |

*Notes:*
Data are presented as unstandardized regression coefficients (β) with standard errors (SE) and standardized coefficients (β).
Dummy coding was used for department, with outpatient department as the reference group.
“—” indicates variables not retained in the stepwise regression model for that outcome.
All variance inflation factor (VIF) values were < 2, indicating no significant multicollinearity.
The results are consistent with those from the main analysis where department was treated as a continuous variable, confirming the robustness of the primary findings.
